# Analysis of Rice Proteins with DLN Repressor Motif/S

**DOI:** 10.3390/ijms20071600

**Published:** 2019-03-30

**Authors:** Purnima Singh, Iny Elizebeth Mathew, Ankit Verma, Akhilesh K. Tyagi, Pinky Agarwal

**Affiliations:** 1 National Institute of Plant Genome Research, Aruna Asaf Ali Marg, New Delhi-110067, India; purnimasingh354@gmail.com (P.S.); inytiny@gmail.com (I.E.M.); vayas06@gmail.com (A.V.); 2 Department of Plant Molecular Biology, South Campus Delhi University, New Delhi-110021, India; akhilesh@genomeindia.org

**Keywords:** EAR motif, rice, seed development, trans-repression, yeast

## Abstract

Transcriptional regulation includes both activation and repression of downstream genes. In plants, a well-established class of repressors are proteins with an ERF-associated amphiphilic repression/EAR domain. They contain either DLNxxP or LxLxL as the identifying hexapeptide motif. In rice (*Oryza sativa*), we have identified a total of 266 DLN repressor proteins, with the former motif and its modifications thereof comprising 227 transcription factors and 39 transcriptional regulators. Apart from DLNxxP motif conservation, DLNxP and DLNxxxP motifs with variable numbers/positions of proline and those without any proline conservation have been identified. Most of the DLN repressome proteins have a single DLN motif, with higher relative percentage in the C-terminal region. We have designed a simple yeast-based experiment wherein a DLN motif can successfully cause strong repression of downstream reporter genes, when fused to a transcriptional activator of rice or yeast. The DLN hexapeptide motif is essential for repression, and at least two “DLN” residues cause maximal repression. Comparatively, rice has more DLN repressor encoding genes than Arabidopsis, and DLNSPP motif from rice is 40% stronger than the known Arabidopsis SRDX motif. The study reports a straightforward assay to analyze repressor activity, along with the identification of a strong DLN repressor from rice.

## 1. Introduction

Regulation of gene expression is an interesting phenomenon with the participation of multiple players, some of which act as repressors of downstream genes and regulate their spatio-temporal expression pattern. Transcriptional repressors act either in an active or passive manner. Active repressors have an intrinsic repression motif which interacts with the general transcription factors (TFs) or with other chromatin components, eventually inhibiting the binding of transcriptional activators [1,2]. TLLLFR, KLFGV, ethylene-responsive element binding factor-associated amphiphilic repression (EAR), and NAC-associated repression domain (NARD) are common plant repression motifs. The ERF–EAR motif contributes about 86% of the total plant repressome in Arabidopsis. Both LxLxL and DLNxxP are EAR motifs [3,4]. Comparatively, LxLxL motif occurs at a higher frequency in different plant species [3]. The EAR repressome is involved in a wide range of functions in plants, from development to stress responses. They regulate plant height [4]; spikelet architecture [5]; flowering time [6]; root hair development [7]; shoot branching [8]; and secondary metabolite biosynthesis [9]. They delimit organ boundaries for proper growth [10]; control floral meristem development [11]; ovule development [12]; and male germ cell division [13]. They prevent expression of stress associated genes under normal conditions, and control growth during stress, and hence save plant energy. Additionally, they cause contained expression of downstream genes, preventing self-damage to the plant [14,15] and imparting a/biotic stress tolerance [16,17]. Some repressors activate plant immunity against pathogens by inducing programmed cell death [18]. Infection by Indian cassava mosaic virus is spread by activation of an EAR motif containing repressor, which suppresses histone methyltransferase [19]. Many EAR repressors also function in hormone signaling pathways, negatively regulating jasmonic acid (JA) signaling [20] and ethylene biosynthesis [21] while positively regulating auxin response [22] and brassinosteroid induced repression [23]. Thus, multifaceted repressors with an EAR motif, many of which have DLNxxP as consensus sequence, eventually cause enhancement or suppression of the downstream response.

A modified EAR motif, called SRDX (modified SUPRD with the sequence LDLDLELRLGFA; SUPRD is a 30 residue C-terminal region of SUPERMAN (SUP) from Arabidopsis, which acts as a repression domain (RD)) and a 32 residue LxLxL-type EAR motif (LDLNLELRISPP) have been successfully used as a dominant repressor in fusion with TFs, to identify the functionality of redundant TFs [24,25]. This technique popularly referred to as chimeric repressor silencing technology (CRES-T) can also be exploited for co-activators, which do not have DNA binding capacity [26]. The LxLxL motif in Aux/IAA (Auxin/Indole-3-acetic acid) proteins is transferable and dominant over activation domains. It functions over a short range and can cause successful repression of an activator, despite the binding site being present either upstream or downstream to the binding site of the activator [27]. The roles of many genes involved in development and stress have been elucidated by using CRES-T [28,29]. Based on the property of transferability of repression motifs, we have designed a yeast-based assay, to easily assess the repressive ability of a TF or motif. In conjunction, the genes coding for transcriptional repressors with a DLN motif in rice have been identified. Their chromosomal localization, expression, and motif patterns have been studied. The residues and length of motif required for repression have been delineated with the aim to discover a strong repression motif from rice. A really strong repression motif can be used for CRES-T, to down regulate downstream genes, especially in cases where only down regulation of a pathway is required, or where the gene cannot be suppressed by standard RNAi technologies due to high homology. An expansion in the basic knowledge of transcriptional repressors from rice can be used for guided genetic modification of the crop, for its improvement in yield and stress resistance.

## 2. Results and Discussion

### 2.1. Rice Has a Huge Repertoire of DLN Transcriptional Repressors

EAR motifs have LxLxL or DLNxxP as the conserved sequence [3]. In this paper, an in silico analysis to identify rice repressors with a DLN motif, showed that rice had 2112 non-transposon proteins containing at least one DLN motif. Out of these, 227 were TFs, belonging to 35 families. Thus, nine percent of the total TFs in rice belonged to the DLN repressome. In Arabidopsis, 6% of the proteome is constituted by TFs, of which 30% are repressors [1]. The maximum numbers of the DLN repressors of rice belonged to C2H2 zinc finger family, with MYB, NAC, AP2 and PHD as other prominent families (Tables S1–S3). We have previously reported the presence of this motif in ZOS TFs [30] and have also shown NAC TFs to act as repressors [31,32]. AP2/ERF repressors having L/FDLNL/F(x)P as the motif essential for repression, were the first ones to be identified [33]. Similar hexapeptide, DLELRL is the minimal motif conferring trans-repression activity to the repression domain of SUPERMAN (SUPRD) Arabidopsis [34], along with the overlapping LxLxL motif [24]. Hence, the most prominent DLN repressor proteins in rice belong to the well-established repressor families.

**Figure 1:**
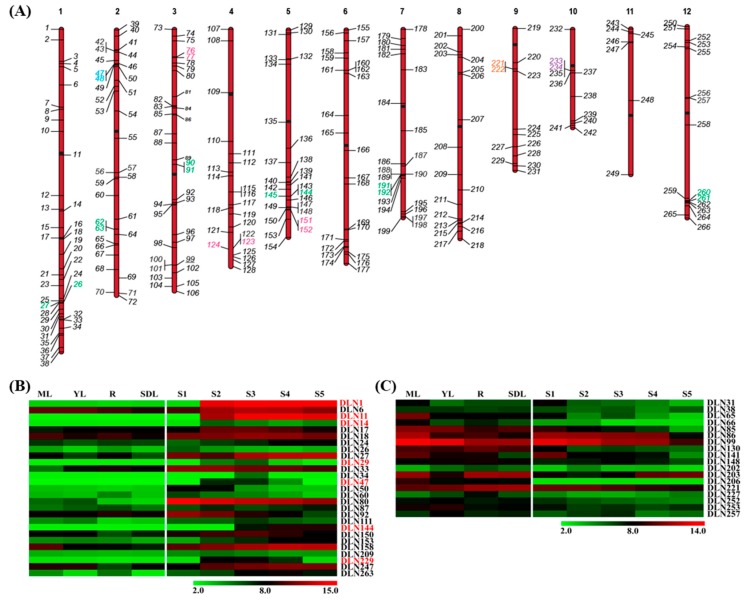
Distribution and expression of DLN repressors encoding genes. (**A**) Chromosomal localization of genes coding for 266 DLN repressors on 12 chromosomes of rice, genes in opposite orientation have been marked on either side and chromosome numbers are mentioned on top. The numbers represent the motif names in the encoded protein. Tandemly duplicated genes have been marked in green, blue, orange, pink, and purple from C2H2, PHD, bZIP, AP2, and btb_poz families, respectively. Heat map has been shown for genes at least two folds significantly (**B**) up- and (**C**) downregulated during rice seed development stages (S1–S5), with respect to vegetative controls (ML—mature leaf, YL—leaf, R—root, SDL—7-day-old seedling). The seed-specific genes have been marked in red in (**B**) and the color legends are shown at the bottom of the heat map.

TFs are localized to the nucleus where they bind to cis-regulatory elements through their DNA binding domains. They can interact with other TFs through oligomerization domains. TFs are categorized into families on the basis of these two domains. They eventually cause either activation or repression of the downstream genes [35,36]. However, TFs alone are not responsible for gene expression. Transcriptional regulators are also indispensable components of the transcriptional machinery. These may/not directly bind to DNA but assist in transcription complex formation by interaction with TFs and other regulators. Prominent regulators are the TBP (TATA-box binding protein) associated factors (TAFs), TBP-related factors (TRFs), mediators, co-activators and co-repressors [37,38]. In our analysis, INTERPRO scan resulted in 39 additional transcriptional regulators, even including basic regulatory elements of transcription (Tables S1 and S3). Amongst the identified regulators, C3HC4 zinc finger proteins were present. These are RING-type zinc finger containing proteins, which participate in transcription by RNA or protein binding [39]. BTB proteins were also identified in our list of transcriptional regulators, and they are well known to function as repressors, and are important for development [40]. Hence, a total of 266 proteins were identified, which can potentially act as DLN repressors in rice. For nomenclature, the motifs were named from *DLN1* to *DLN266*, after being arranged in the order of chromosome numbers and their positions. Most of the identified repressors had a single DLN motif, while only 14 had two motifs, making a total of 280 identified motifs. Two motifs of the same gene were given the same name with a or b as the suffix for the first and second motif, respectively (Table S1). The genes encoding for these were found to be localized on all 12 chromosomes of rice. Tandem duplication was seen amongst 24 repressors belonging to C2H2, PHD, AP2, bZIP, and btb_poz family (Table S4, Figure 1A). The identification of repressors even in transcriptional regulatory proteins, in both rice and Arabidopsis, points to the relevance of this motif in conferring functional properties [3,41]. Since repressors are important for reproductive development of the plant [8,9,10,11,12,13,28,34] and we have been examining the developmental biology of the rice seed [35,42,43,44], the expression of the rice DLN repressors was studied in five stages of rice seed development [35], with respect to four vegetative controls by microarray analysis [44]. There was significant up- and downregulation of 27 and 18 genes, respectively, whose encoded protein had a DLN repressor motif. Out of these, seven genes were seed-specific, including motif DLN144 from *ZOS5-09* (Figure 1B,C). *ZOS5-09*, as reported previously by us, is a typical C_2_H_2_ zinc finger gene, and expresses at extremely high levels in rice seed [30]. Since analysis of transcriptome of rice is being exploited for crop improvement in terms of yield [45,46,47], repressors with such specific expression should be examined in detail to determine if they expedite or slow down the developmental process.

**Figure 2:**
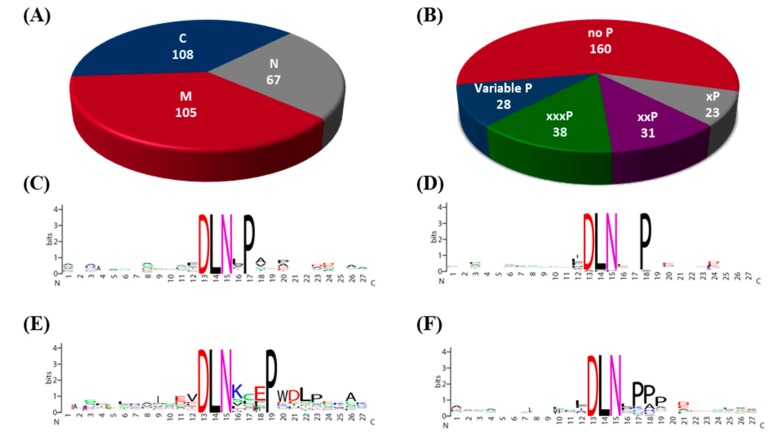
DLN motif analyses. Pie charts showing (**A**) distribution of DLN motifs in different regions of the protein, namely N-terminal (N), middle region (M), and C-terminal (**C**), and (**B**) the numbers of four types of consensus DLN motifs found in rice, with conserved residues, determined by WebLogo, as (**C**) DLNxP, (**D**) DLNxxP, (**E**) DLNxxxP, and (**F**) variable P.

These motifs were found to be located all over the protein, i.e., at the N- and C-termini and middle regions. Though C-terminal constitutes only 25% of the protein, maximum numbers of motifs were found in this region (Figure 2A). Two DLN motifs, wherever present, were located in adjacent regions (Table S3). DLN73 was an exception, with the motifs a and b being spaced out in the N- and C-terminal regions. DLN85 and DLN147 were located on two MYB TFs, with both the motifs in the C-terminal region. Till date, a large number of proteins have been identified with the DLN motif being mostly at the C-terminal of the protein [13,21]. The wide distribution of the DLN repressor motif, is an indication of the essentiality and flexibility of repressor proteins. The consensus sequence, spanning 27 residues surrounding the motif indicated that there was conservation of DLNxP, DLNxxP, DLNxxxP, and variable P, unlike only DLNxxP in Arabidopsis [3]. Out of a total of 280 motifs identified in this study, 160 motifs did not have conservation of any other residue, except DLN, as shown by WebLogo analysis (Figure 2B–F, Tables S1 and S3).

### 2.2. DLN Motif Is a Strong Repressor

**Figure 3:**
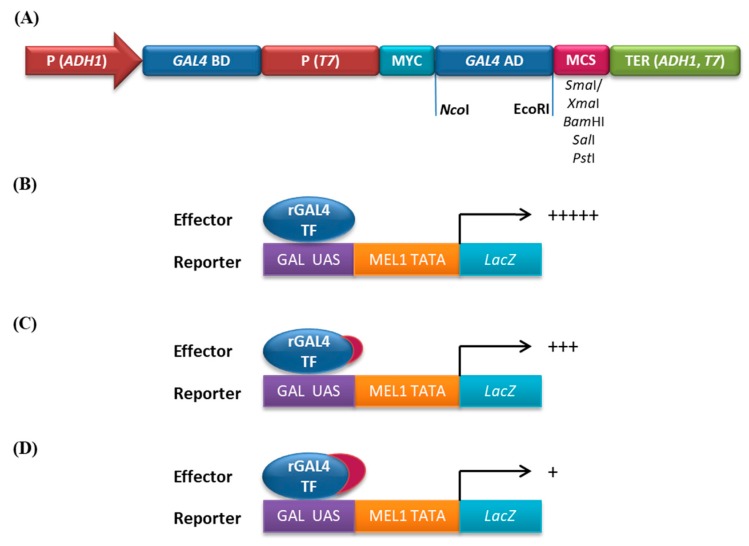
Reconstitution of GAL4 TF and repression by DLN motif. (**A**) Line diagram showing cloning of activation domain of GAL4 TF (GAL4 AD) in the pGBKT7 vector to form the rGAL4 vector. The partly reconstituted GAL4 TF is called as rGAL4; The multiple cloning site (MCS) was used for cloning genes/segments; (**B**) rGAL4 bound to the UAS of *LacZ*, was a strong activator, which could be quantitated using ONPG as a substrate. (**C**) Repressor (red) fused with rGAL4 decreased *LacZ* expression and (**D**) a strong repressor (red) drastically reduced reporter gene expression, as indicated by “+” signs.

Repression motifs are “portable”, implying their full functionality on heterologous DNA-binding domains [48]. In conjunction, DLN motif fused with the VP16 activation domain or GAL4 (galactose) DNA-binding domain can greatly suppress the activity of a reporter gene caused by a minimal promoter [33]. We have used this premise to assess the repressive ability of the DLN motif, in a simplified trans-repression assay in yeast, as reported recently by us [32]. For this, the GAL4 transcription factor was reconstituted (rGAL4), by fusion of the activation domain/AD to the DNA-binding domain/BD in the vector pGBKT7, resulting in formation of rGAL4 vector (Figure 3A). The resulting rGAL4 caused transactivation of the *LacZ* reporter gene in yeast strain AH109. The GAL4 BD bound to the UAS in the promoter of this gene, while the activation domain was responsible for the gene activity, which could be recorded and quantitated (Figure 3B). On fusion of r*GAL4* with a repression motif, this activity was significantly decreased, depending on the repression ability of the motif (Figure 3C,D). Generally, TFs are shown to be trans-activators in yeast [49]. Often a TF or motif is categorized as a repressor, on the basis of its inability to transactivate yeast reporter genes [50]. Otherwise, a repressor function was demonstrated by reporter-effector assays in plant protoplasts where the effector and the domain to which it will bind are cloned in separate constructs, and the reporter activity is relatively quantified [11]. To avoid the complicacy of this assay, we have used rGAL4-DLN fusions in our trans-repression assays and showed them to be effective [32].

**Figure 4:**
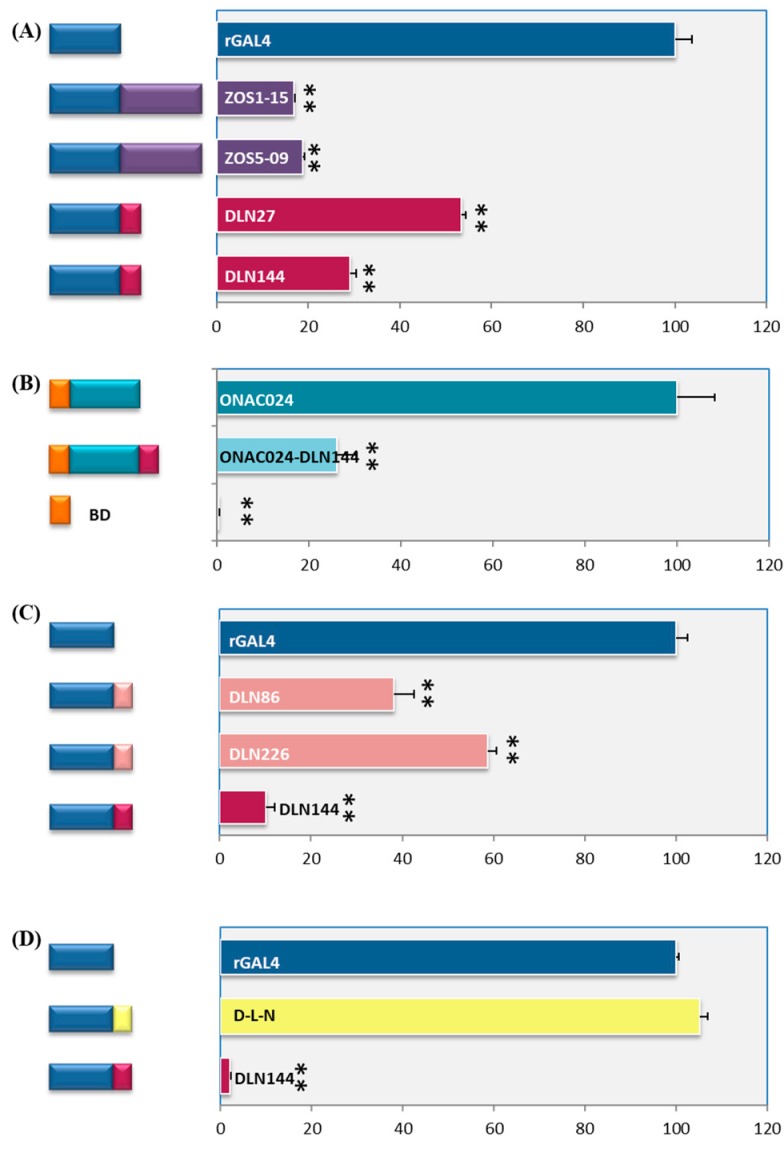
Repressive ability of the DLN motif. ONPG assay showing β-galactosidase units, with o-nitrophenyl-β-d-galactopyranoside as the substrate, on the *x*-axis for various fusion products as mentioned. (**A**) rGAL4 fused with complete ORFs (purple bars) of ZOS1-15, ZOS5-09 and their DLN motifs (red bars), DLN27 (DLNYPP), DLN144 (DLNSPP), respectively; (**B**) DLN144 fused with an activator, ONAC024; (**C**) DLN86 and DLN226a lacking P conservation in the hexapeptide, fused with rGAL4, and (**D**) the activity of tripeptide D-L-N motif in comparison with the hexapeptide DLN. The repression efficacy of all constructs has been depicted in terms of percentage in comparison to the transactivation ability of reconstituted GAL4 TF (rGAL4, in **A**, **C** and **D**). In (**B**), DLN144 hexapeptide motif from ZOS5-09 represses activation by ONAC024, and the binding domain (BD from vector pGBKT7) is the negative control. The line diagrams on the left side are a representation of the fused protein, with blue and red colors denoting rGAL4 and DLN144 hexapeptide motif from ZOS5-09, respectively. The asterisks represent *p*-value ≤ 0.005, in biological triplicates. The standard error bars have been marked.

We earlier reported that the proteins coded by seed-high genes *ZOS1-15* and *ZOS5-09* have a DLN motif [30]. These genes do not exhibit trans-activation in yeast. For trans-repression assays, the ORFs of these genes were fused downstream to rGAL4 and the activity of the reporter *LacZ* gene was analyzed by quantitative ONPG assay. It emerged that both of them significantly repressed rGAL4 activation by 80%. To test if the repression by ZOS1-15 and ZOS5-09 was due to their DLN motifs, DLN27 and DLN144, respectively were fused with rGAL4 and reporter activity was assessed. Their hexapeptide DLN motifs also proved to be repressors, with DLN144 from ZOS5-09, being stronger (Figure 4A). Hence, we used this motif (DLN144) for further experiments. In order to assess the efficacy of this motif, a strong transcriptional activator, namely ONAC024 from rice, was fused with DLN144. This resulted in a 70% reduction of the activation by ONAC024 (Figure 4B). To further strengthen these results, similar repression was observed on X-gal chromogenic substrate containing media (Figure S1A,B). Additionally, DLN144 motif proved to be so strong that it even slowed the growth of yeast cells (Figure S2). Overexpression of DLN repressors often affects plant growth [51] and GroupVIII ERFs induce cell death [52]. We have hypothesized that the growth is affected because of their probable repression of the cell cycle upon interaction with histone deacetylases [32]. Since these two, along with the minimal repression motif from SUPERMAN, i.e., DLELRL [34], are repressors, it seems that the presence of residues subsequent to DLN is essential for maintenance of the amphiphilic property of the hexapeptide, and may/not essentially contain proline. So, we examined the repressor activity of DLN sequences lacking the conserved P residue in the hexapeptide sequence, namely, DLN86 and DLN226a with DLNKCE and DLNSID motifs, respectively. They also significantly repressed activation by rGAL4, though they were not as strong as DLN144 (Figure 4C, Figure S1C). This indicated that DLN hexapeptides without proline can also cause repression. The next imperative analysis was to examine if the entire hexapeptide was essential for repressive activity. For this, only “DLN” tripeptide was fused to rGAL4 and reporter gene activity was analyzed. Just the tripeptide DLN was not able to cause any repression of rGAL4 (Figure 4D, Figure S1D), implying the requirement of the entire hexapeptide for the repressive ability of this motif. Another new motif identified in this study was DLNxxxP (Figure 2B,E). We have recently shown that ONAC020 and ONAC026 with DLN1 and DLN11 motifs, respectively, are strong repressors. Both have the sequence DLNKCEP, which is xxxP type of motif [32]. Further analysis done in our laboratory shows that this motif contributes to the repressive property of these two genes (unpublished data). Both DLN27 and DLN144 are xPP, i.e., variable P type of motif (Table S1, Figure 2). Therefore, we can hypothesize at this juncture that the various types of DLN motifs reported in this paper should act as potential repressors. In earlier reports, the DLN motif stretch of TFs has been found critical for their activity [21]. The EAR motif has been found to be essential for protein–protein interactions, especially for proteins interacting with co-repressors TPL/TPR. The co-repressors interact with histone deacetylase causing repression [10,23,53]. Therefore, an alteration in the hexapeptide probably changes its interaction property, decreasing its repressive ability. Hence, the putative DLN repressor motifs identified in this study may be able to repress downstream genes. However, the efficacy of each motif should be tested by a trans-repression assay, before using it for further experimentation.

### 2.3. At Least Two Amino Acids Should Intact for Maximal Repressive Activity

**Figure 5:**
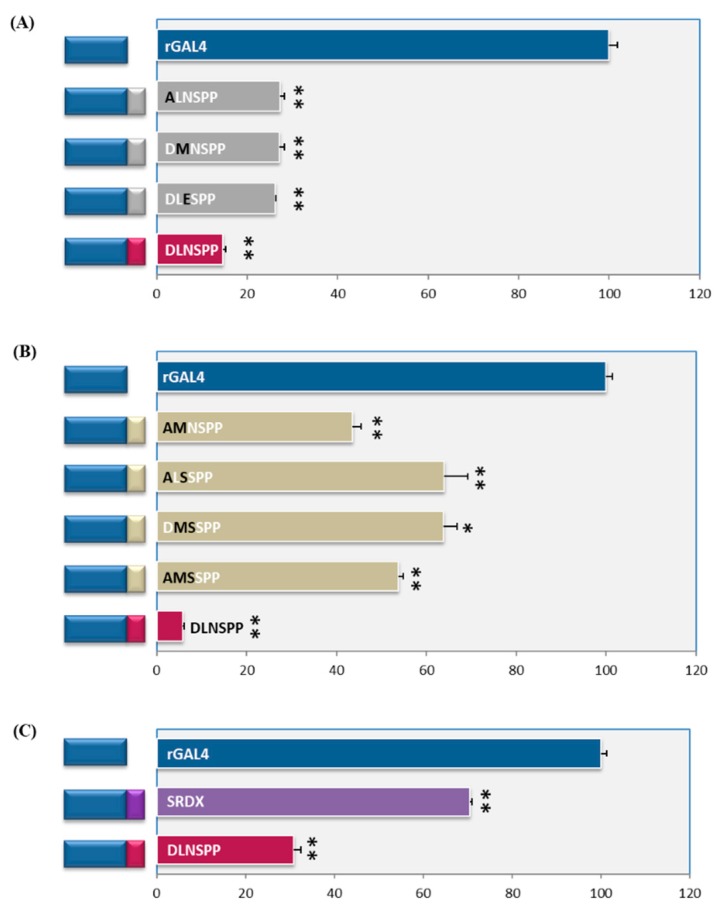
Mutation analysis of hexapeptide DLN144 motif (DLNSSP) and comparison with SRDX. The bar graphs show results of ONPG assay depicting β-galactosidase units on the *x*-axis, as a percentage of activation by rGAL4, for (**A**) single, (**B**) double, and triple mutations of DLN. All mutated residues are indicated in black; (**C**) comparison of the repressive abilities of SRDX from Arabidopsis and DLN144 motif from rice when fused with rGAL4 activator. In all figures, the pictorial representation of the fused protein is on the left side. rGAL4 is represented in blue color and the DLN hexapeptide in red color. Double and single asterisks represent *p*-values of ≤0.005 and ≤0.05 in biological triplicates. The standard error bars have been indicated on the bar graphs.

The DLN motif has alternate hydrophilic and hydrophobic amino acids, with aspartic acid being amphiphilic, and hence the name EAR or ERF-associated amphiphilic repression motif. In Arabidopsis, hydrophilic D on being replaced by hydrophobic A, results in significant loss of repressive activity [33]. A minimum of six residues (DLELRL) are required for successful repression by SUPRD, with at least two leucine residues intact. The hydrophilic nature of the residues is also essential for proper repression activity [34]. In case of Aux/IAA proteins, loss of leucine or threonine residues results in drastic reduction in repression [27]. To test the efficacy of the DLN residues in the rice hexapeptide of DLN144, single, double, and triple mutations were made in D, L and N residues. The mutated residues were fused downstream of rGAL4 and reporter gene activity was assayed. There was only 12% loss of repressive activity when a similar mutation was above was made, with D replaced by A. Other single residue replacements, in which the property of the amino acid was maintained (L replaced with M, both hydrophobic and N replaced with E, both hydrophilic) also showed comparable results (Figure 5A, Figure S1E). Further, double and triple mutations were made in the DLN motif. All mutations were kept the same as above, except N was substituted with S, a neutral amino acid. These substitutions caused 37–57% loss of repressive activity of DLN144 hexapeptide (Figure 5B, Figure S1F). Since complete abolishment of repression was not observed and the three residues by themselves do not act as repressors (Figure 4D); it is highly probable that amphiphilic property of the hexapeptide [33] is responsible for repression, and at least two residues of “DLN” have to be present for maximal repression to occur. The importance of “DLN” in the hexapeptide is reiterated by the fact that functionally, ERF3 does not interact with TPL when “DL” is mutated [54], mutation of “D” in Gm-JAG1 to H results in narrow leaflet varieties [55] and of L to A abolishes repressive property in OsERF3 [21], and ZAT7 [56].

### 2.4. There Are Subtle Differences amongst DLN Motifs from Rice and Arabidopsis

The Arabidopsis genome has a total of 219 EAR motif-containing transcription related proteins, including both DLNxxP and LxLxL type motifs. Out of these, 75 proteins contain a DLNxxP motif, distributed amongst 13 TF families [3]. In our analysis, we identified a total of 227 DLN motif containing TFs in rice, indicating an amplification of this repressor protein, and diversification into TF families not containing this motif in Arabidopsis. In addition, eight transcriptional regulatory proteins contain a DLN motif in Arabidopsis, while the number has increased to 39 in rice. The C2H2 family both from Arabidopsis and rice is the largest with 29 members in Arabidopsis and 41 in rice containing DLN motif. Also, the AP2/ERF family has considerable DLN repressor proteins in both plants. Most of the DLNxxP motifs (64%) are present at the C-terminal end of the proteins in Arabidopsis, with few at N-terminal (27%) and middle segment of the protein (9%) [3]. On the contrary, in rice, the EAR motif was distributed almost equally at the C-terminal (38.5%) and middle (37.5%) regions. Less than one fourth of the motifs were present at the N-terminal end. Moreover, the proline residue at the sixth position in the DLNxxP EAR motif was absolutely conserved in Arabidopsis, unlike rice (Figure 2).

The SRDX motif (LDLDLELRLGFA), which is used for CRES-T [25], causes up to 30% repression of the target genes [26]. Hence, we tested the repressive ability of the SRDX motif in the rGAL4 vector. SRDX repressed rGAL4 activation of reporter genes up to 30%, strengthening our claim that yeast rGAL4-repressor motif fusion can be effectively used for tran-srepression assays. Interestingly, DLN144 proved to be a stronger repressor, and resulted in 70% repression in the same experiment (Figure 5C, Figure S1G and S2). Hence, the DLN motif of ZOS5-09, i.e., DLN144 can be used for strong repression of genes downstream to a TF. Using CRES-T, male and female sterile plants can be created for hybrid generation [57] and photoperiodic pathways can be altered, which can be used for agronomic purposes [58]. Hence, the repressors can be exploited not only for functional studies but also for crop improvement.

## 3. Materials and Methods

### 3.1. Data Mining

Protein database of rice pseudomolecule version 7.0, at MSU (Rice Genome Annotation Project, http://rice.plantbiology.msu.edu/cgi-bin/ORF_infopage.cgi) was used to extract all proteins with the residues “DLN”, using in-house formulae and VBA macros in Microsoft^®^ Excel. From these, TFs were identified by comparison with those listed in our previous analysis [44], PTFD (Plant Transcription Factor Database v3.0) [59] and DRTF (Database for Rice Transcription Factors) [60]. Further, the rest of the proteins were analyzed in INTERPRO, to determine the transcriptional regulators with a DLN motif. The genes coding for the DLN repressome were sorted according to their chromosome numbers and position and the motifs were named accordingly. Two motifs on the same protein, wherever present, were named as “a” and “b”, respectively. The positions of all elucidated genes were marked on 12 chromosomes of rice using MapChart 2.3 (https://www.wageningenur.nl/en/show/Mapchart-2.30.htm), based on the CDS co-ordinates obtained from MSU. Also, tandemly duplicated genes were elucidated as previously described [30]. The DLN motif sequence was of a total of 27 residues, spanning 12 residues either side of DLN [3]. Only in cases where DLN was present at extreme ends, was the motif smaller in length. The consensus sequences for the motifs were generated in WebLogo (http://weblogo.berkeley.edu/logo.cgi), using default parameters. For determination of the motif location, the first and the last 25% of the residues of the total protein sequence were assigned to the N- and C-terminal regions, respectively, while the rest of the residues belonged to the middle region [3]. The significantly (*p*-value ≤ 0.05) up- and downregulated genes, by at least two folds, during seed development stages [35], in comparison to four vegetative controls, were identified as previously described [45].

### 3.2. Construction of Effector Plasmids

The complete ORF of genes coding for selected TFs/segments were cloned downstream of a partly reconstituted GAL4 TF (rGAL4), and these acted as effector plasmids. The yeast GAL4 TF was partly reconstituted by cloning 342 bp coding for the activation domain (AD), in pGBKT7 vector (Clontech laboratories, Inc., Mountain View City, CA, USA) between *Nco*I and *EcoR*I restriction sites, downstream of the binding domain (BD) and in-frame to it (Figure 3A). Thus, BD and AD were cloned in the same vector, which resulted in the formation of a strong transcriptional activator, rGAL4 and caused strong expression of endogenous reporter gene *LacZ* in yeast strain AH109 (Clontech laboratories, Inc.), which was under the control of GAL4 responsive upstream activating sequence and MEL1 TATA promoter (Figure 3B). When AD was cloned out of frame with BD, functional rGAL4 was not reconstituted, and hence reporter gene did not express.

For analyzing the repressive activity of proteins with a DLN motif, complete ORFs of *ZOS1-15* (LOC_Os01g62190) and *ZOS5-09* (LOC_Os05g38600), were cloned in-frame between *EcoR*I and *BamH*I restriction sites, downstream to rGAL4. To assess the repression ability of different DLN motifs and their mutated forms (DLN144 from ZOS5-09 and DLN27 from ZOS1-15; motifs without P conservation in the hexapeptide, DLN86 from ONAC039 (LOC_Os03g21030) and DLN226a from ZOS9-15 (LOC_Os09g29750); three residue DLN, SRDX from Arabidopsis [25] and DLN144 with single, double and triple mutations), single-strand forward and reverse linear oligos with *EcoR*I and *BamH*I restriction sites were chemically synthesized (Table S5). The complementary oligos for each construct were annealed at 25 °C for 45 min to obtain double stranded oligos with 5′ *EcoR*I and 3′ *BamH*I restriction overhangs. These motifs were subsequently ligated at 25 °C for 5 min, between these restriction sites, downstream to rGAL4. Additionally, to check the activity of hexapeptide DLN144 from ZOS5-09 in the presence of another known activator, the CDS of *ONAC024* was fused in frame with DLN144, by amplification, using 5′ gene-specific primer with *Nco*I restriction site and 3′ gene-specific primer with DLN144 coding sequence and *Sal*I restriction site. The amplicon was ligated between the corresponding sites in pGBKT7 vector.

### 3.3. Analysis of Repressive Activity of the Constructs

The effector constructs were transformed in yeast strain AH109 by using EZ-Yeast^TM^ transformation kit (MP Biomedicals), according to the manufacturer’s protocol and were selected on SD/-Trp selection plates (synthetic drop out plates, without tryptophan). The positive transformants were used to perform β-galactosidase based qualitative and quantitative assays to check the activity of the effector constructs. For quantitative ONPG (o-nitrophenyl β-d-galactopyranoside) assay, three independent colonies as biological replicates and three technical replicates for each colony, were used for each effector construct. ONPG assay was performed according to the yeast protocol handbook (Clontech laboratories, Inc). The β-galactosidase activity was calculated using the formula—β-galactosidase units = (1000 × OD_420_)/(t × V × OD_600_) where t is elapsed time of incubation; V is 0.1 mL x concentration factor. The statistical significance of the results was tested by conducting an F-test for two sample variance followed by student’s *t*-test (assuming equal or unequal variances, as resulting from F-test) in Microsoft Excel^®^ with hypothesized mean difference 0 and α = 0.05. Similarly, X-gal (5-bromo-4-chloro-3-indolyl-β-d-galactopyranoside) was used as the substrate for qualitative assay. Positive colonies for each effector construct were patched on SD/-Trp media, containing 80 mg/L X-gal and were incubated at 30 °C, for two to three days, in dark. Also, yeast drop out assay was performed to study the effect of DLN144 from ZOS5-09 and SRDX on the growth of yeast cells. The initial culture was grown up to 0.2 at OD_600_ and was serially diluted in 1:2 ratios. These were spotted and incubated on SD/-Trp plate and photographed.

## 4. Conclusions

In the rice genome, 266 genes code for proteins (including 227 TFs and 39 regulatory proteins) which contribute the DLN repressome. They are distributed uniformly over 12 rice pseudomolecules and seven are specific to seed development stages. The majority of the proteins had a single motif, with more relative numbers in middle and C-terminal regions of the proteins. Though a few motifs had conserved residues such as xP, xxP, and xxxP, those without any such proline conservation (DLNSID and DLNKCE) or variable proline conservation in the hexapeptide (DLNSPP and DLNYPP) also acted as repressors. A six residue DLN motif from ZOS5-09 (DLN144) caused at least 70% (going up to 98% in some experiments) repression of the reporter gene *LacZ*. While alteration of at least two residues of DLN, significantly reduced the repressive ability, only “DLN” as a tripeptide did not cause any repression. The motif was a stronger repressor than the commonly used repressor motif from Arabidopsis, SRDX, and can be used for effective downregulation of downstream genes, both for basic and agricultural purposes. Moreover, the rGAL4 vector can be successfully used for analyzing repressive ability of TFs/domains in simple trans-repression assays in yeast.

## Supplementary Materials

It could be found at https://www.mdpi.com/1422-0067/20/7/1600/s1.
